# Contribution of Sensory Function to Preclinical Indicators of Physical and Cognitive Functioning With Aging

**DOI:** 10.1093/geroni/igab046.1695

**Published:** 2021-12-17

**Authors:** Yuri Agrawal, Jennifer Schrack, Bonnielin Swenor

## Abstract

There are well established associations between sensory loss and physical and cognitive deficits with aging, but gaps remain in our understanding of the associations between sensory function and early preclinical indicators of physical and cognitive decline. This symposium will present data from the Baltimore Longitudinal Study of Aging (BLSA) on a series of studies investigating the links among sensory function, motor function, and physical and cognitive outcomes in older adults. In the first study, Dr. Gross will present an operational definition of early cognitive impairment (ECI) based on a combination of two cognitive measures – the Card Rotations test and the California Verbal Learning Test Immediate Recall – to predict progression to MCI/AD. In the second study, Dr. Cai will evaluate the relationship between multisensory impairment (in vision, hearing, olfaction, proprioception and vestibular function) and the algorithmic definition of ECI. In the third study, Dr. Armstrong will evaluate the association between multisensory impairment and another biomarker of ECI or preclinical AD, specifically PET-PiB deposition. In the fourth study, Dr. Schrack, will present the joint contribution of multisensory (hearing and vision) impairment and motor function (gait speed) on risk of incident MCI/AD in longitudinal analyses. Finally, Dr. Martinez Amezcua will present the longitudinal association between hearing and vestibular function and decline in higher level physical function and endurance performance. Taken together, these studies present compelling data about the contribution of sensory function to preclinical indicators of physical and cognitive functioning with aging.

